# Checklist of the micromolluscs in the intertidal zone of the Yellow Sea and Bohai Sea, China

**DOI:** 10.3897/BDJ.11.e105444

**Published:** 2023-07-07

**Authors:** Lu Qi, Biyang Xu, Lingfeng Kong, Qi Li

**Affiliations:** 1 Key Laboratory of Mariculture, Ministry of Education, Ocean University of China, Qingdao, China Key Laboratory of Mariculture, Ministry of Education, Ocean University of China Qingdao China; 2 Sanya Oceanographic Institution, Ocean University of China, Sanya, China Sanya Oceanographic Institution, Ocean University of China Sanya China; 3 Laboratory for Marine Fisheries Science and Food Production Processes, Laoshan Laboratory, Qingdao, China Laboratory for Marine Fisheries Science and Food Production Processes, Laoshan Laboratory Qingdao China

**Keywords:** biodiversity, description, mollusca, morphology

## Abstract

**Background:**

The diversity of the sedimentary environment and molluscs is rich in the intertidal zone of the Yellow Sea and Bohai Sea. At present, many of the investigations focus on macromolluscs, while the diversity of micromolluscs is seriously underestimated.

**New information:**

In this study, the survey of micromolluscs was carried out in the intertidal zone of the Yellow Sea and Bohai Sea. The collection and preservation of micromolluscs, as well as the preparation methods of morphological characteristic structures by scanning electron microscopy (SEM) were explored. A total of 20 species were described in this survey. These can be assigned to 16 families, four orders (Vetigastropoda (1), Caenogastropoda (10), Heterobranchia (5) and Autobranchia (3)) and two classes (Gastropoda (17), Bivalvia (3)).

## Introduction

Molluscs are one of the largest groups of marine benthos, with a huge variety of life strategies. In marine ecosystems, molluscs occupy different trophic levels, from filtrators and phytophages to predators and parasites. Due to their high diversity and widespread distribution, they have been widely recorded in taxonomic monographs. Recent examples of taxonomic monographs from the Western Pacific Region included Liu (2008), Zhang et al. (2016), Okutani (2017), and Li et al. (2019). These monographs recorded a large number of taxa, but most of them are macromolluscs, while micromolluscs are not fully understood, especially in China.

Micromolluscs are important components of molluscs and play an important role in biodiversity assessments. As micromolluscs usually require specific collecting/sorting attention and have a reputation for presenting formidable taxonomic difficulties, they are often overlooked in biodiversity surveys, resulting in a gross underestimation of mollusc diversity. In a biodiversity survey of New Caledonia, the micromolluscs account for approximately one-third (33.5%) of all identified mollusk species, with the largest size class being 1.9–4.1 mm (Bouchet et al. 2002). Therefore, it is of great significance to carry out micromolluscs' surveys to assess the diversity of molluscs.

The diversity of the sediment environment and molluscs is rich in the intertidal zone of China. However, much of the literature focuses on macromolluscs that can be collected by hand-picking in the field. Only Liu (2013) investigated the small gastropods in the *Zosteramarina* in Swan Lake, Rongcheng Bay China. Here, we carried out the survey of micromolluscs in the intertidal zone along the coasts of the Yellow Sea and Bohai Sea, established methods for sample collection and preservation, used morphological and molecular data to identify the species and performed morphological descriptions based on SEM.

## Materials and methods

### Taxon sampling and processing

**Figure 1.**
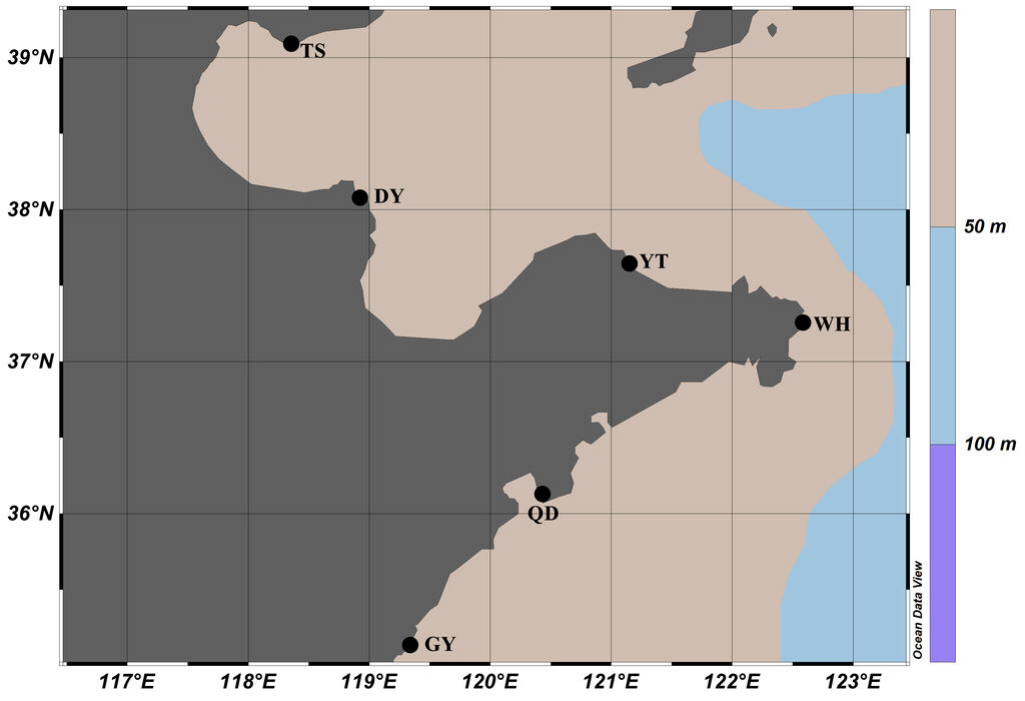
Map of sampling site. For details on collection sites, see Table 1.

Sampling was carried out in the intertidal zone of the Yellow Sea and Bohai Sea from September 2017 to December 2019 (Fig. 1, Table 1). Sample collection was performed with reference to the handbook by Li et al. (2019). Field collection of micromolluscs requires some specialised techniques depending on the likely habitats of the target organisms and a range of microhabitats.

Shell grit or shell sand

The sediments usually contain mainly empty shells and certain species should be divided into size fractions by using graded sieves (e.g. 10, 5, 2.5, 1.0 and 0.4 mm mesh size). If there is a need to collect all adult species, the 0.4 mm sieve is suitable.

Algal samples

Algae are one of the most important habitats for micromolluscs. After the algae are collected on-site, it should be placed in a bucket or ‘zip-lock’ bag and then the bucket or bag should be shaken violently. The algal material is then removed and the sample is allowed to settle briefly. The water can then be gently decanted, being run through a sieve to catch any floating molluscs.

Rock

The upper and undersides of rocks are very different environments. Some micromolluscs may be attached to the algal films or turf on the rock surface. These rocks can be scrubbed with a brush in a bucket. Then the residue in the bucket is collected into the sample bottle.

Specimens for anatomical study were stored in formalin, whereas for molecular work, strong (> 95%) ethanol or RNALater are the best preservatives. All samples were brought back to the laboratory for further processing. All the material collected has been deposited in the Laboratory of Shellfish Genetics and Breeding (LSGB), Fisheries College, Ocean University of China, Qingdao, China. Morphological identification of the species was carried out mainly through the identification book from Okutani (2017) and original literature, as well as WoRMS (http://www.marinespecies.org).

### Molecular analyses

Traditional barcoding gene COI was analysed for most specimens to verify identification. Total genomic DNA was extracted from entire animals with the TIANamp Marine Animals DNA Kit (Tiangen Biotech, Beijing, China), according to the manufacturer’s protocol and stored at -4°C for short-term use. Amplification of partial sequences of mitochondrial COI was amplified by a polymerase chain reaction using the primers from Folmer et al. (1994), LCO1490 (5'-GGTCAACAAATCATAAAGATATTGG-3') and HC02198 (5'-TAAACTTCAGGGTGACCAAAAAATCA-3'). PCR reactions were made in a volume of 25 μl, using the following: 19.3 μl distilled H_2_O (sterile MilliQ), 0.5 μl dNTPs (2.5 mM), 2.5 μl 10×buffer（Mg^_2_+^ plus）, 0.2 μl TakaRa Taq DNA polymerase, 1 μl of each primer (10 μM) and 0.5 μl genomic DNA (50-100 ng). Thermal cycling conditions were: 94℃ for 3 min, followed by 37 cycles of 94℃ for 45 s, 48℃ for 45 s, 72℃ for 60 s and a final extension of 72℃ for 10 min. PCR products were run in a 1.5% agarose gel to corroborate the success of the amplifications and sequenced by Shanghai Bioengineering Co., Ltd (China). DNA sequences were assembled using SeqMan (Swindell and Plasterer 1997). The obtained sequences were compared to sequences of molluscs in both GenBank and BOLD databases. Sequences have been deposited in GenBank (Table 2).

### Preparation of micromollusc shells for SEM

Specimens can be immersed for one to two minutes in strong commercial bleach to remove the periostracum. The soaked shells were placed in a centrifugal tube with neutral detergent and cleaned by ultrasound. The cleaning time depends on the amount of dirt on the shell surface. After sonication, shells should be washed in distilled water preferably two or three times. After the last wash, the water drops should be removed with a paper towel and the specimen air-dried. The clean shells were attached to conductive glue in the direction of the standardized views and then sputtered with gold. The thickness of the metal coating is 1-10 nm; however, even excessive coating will not interfere with the detail in a normal shell. The operating tool was TESCAN VEGA3 scanning electron microscope (SEM).

## Checklists

### Checklist of the micromolluscs in the Yellow Sea and Bohai Sea, China

#### Lirularia iridescens

Schrenck, 1863

60F1397C-B0E8-5774-AB81-1F521439996D

##### Native status

Found on seagrass between tidal and shallow waters.

##### Distribution

Yellow Sea of China; Japan Sea, South Kurile Islands.

##### Notes

*L.iridescens* was recorded by Kantor and Sysoev (2006). Okutani (2017) described and illustrated in detail *L.iridescens*. In this study, the species was re-described, based on the SEM. Crater‐like pitted microsculptures on the protoconch were first discovered here. At present, there are very few morphological studies on this genus *Lirularia*. Many studies have shown that *L.iridescens* are phytal gastropods inhabiting seaweeds (Toyohara et al. 1999, Agatsuma et al. 2005).

##### Diagnosis

**Figure 2.**
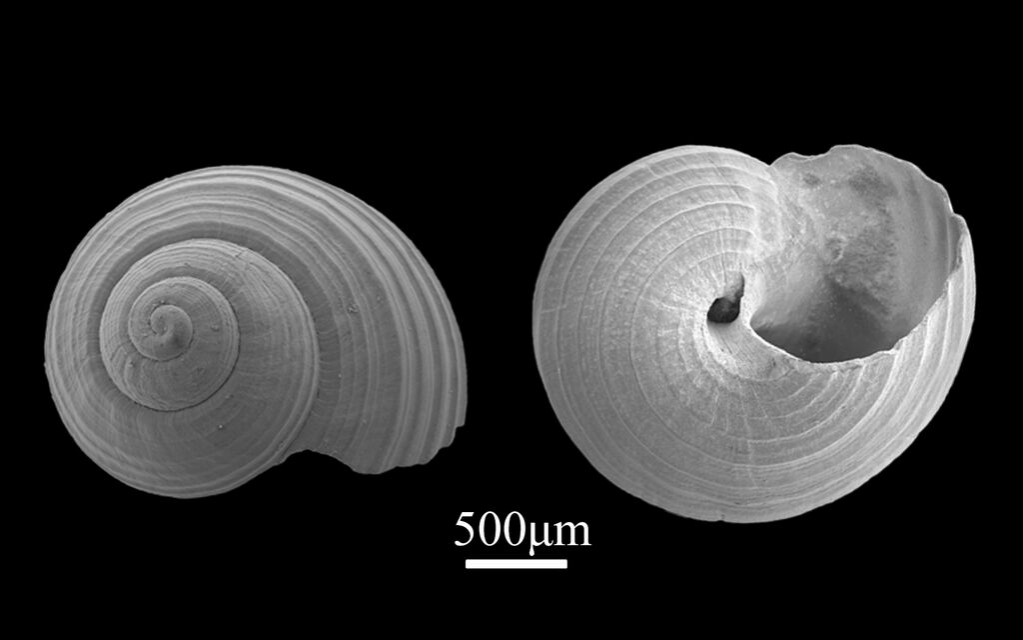
*Lirulariairidescens* Schrenck, 1863.

Shell minute (2.5±0.26 mm in width, 2.2±0.15 mm in length), ovato-conica. Whorls 4-5, spire low, body whorl rather ventricose (Fig. 2). Suture distinct. Every whorl is striated by about 9-10 distinct spiral ribs and the distance between the spiral ribs is not uniform. Reddish-brown spiral ribs contrasting with iridescent interspaces; lines on ribs occasionally broken by white spots, with crater‐like pitted microsculpture on protoconch. Aperture ovate, outer lip thin, with small notches, inner lip simple and slightly convex. Umbilicus circular deep. Basal surface flat, with 9-10 circular spiral ribs.

#### Alaba picta

A. Adams, 1861

55FF3334-B96A-53C7-B87A-869FF9BFE8A4

##### Native status

Found on seagrass between tidal and shallow waters.

##### Distribution

From Liaoning to Shandong coasts of China; Japan; Australia.

##### Notes

*Alabapicta* with obvious morphological variance in colour, aperture, shell length and sculpture (Liu 2013). *Dialavitrea* G. B. Sowerby, 1915 is the name given to a phenotype lacking a colour-pattern (Okutani 2017). *Dialapicta* A. Adams, is the original name of *A.picta*, which is from the Shandong coast of China (Adams 1861).

##### Diagnosis

**Figure 3.**
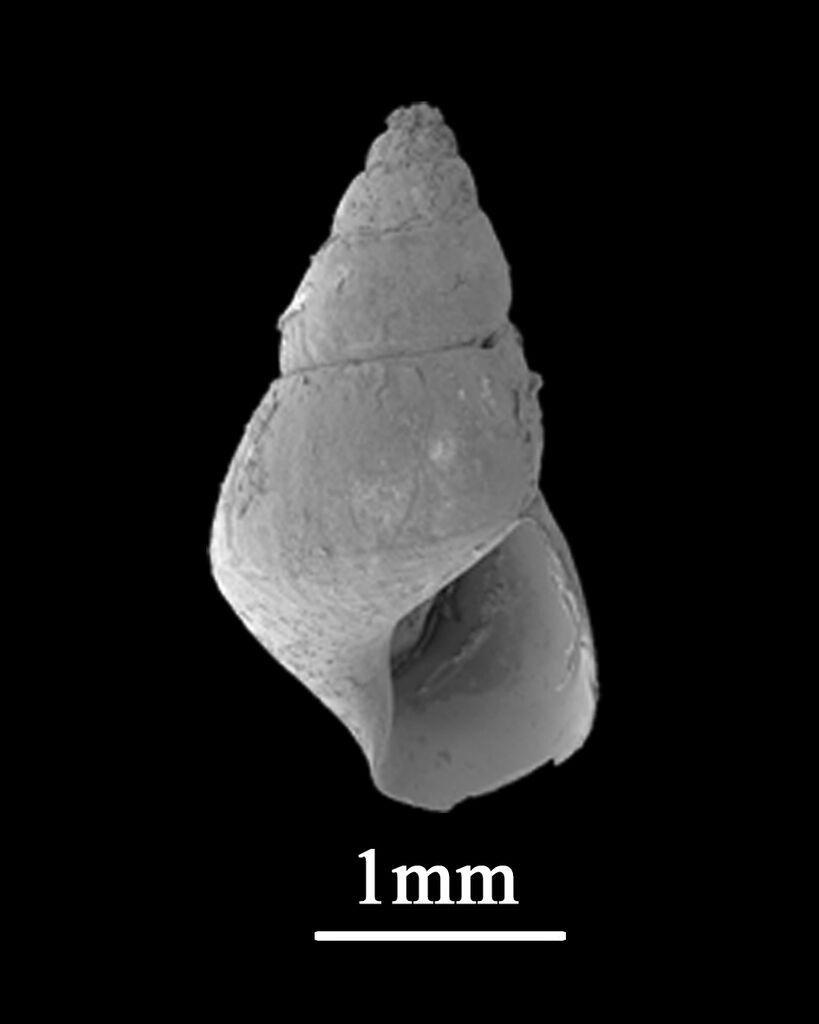
*Alabapicta* A. Adams, 1861.

Shell small (8.5±0.34 mm in height, 4.5±0.18 mm in width), ovate-conica, thin, fragile, translucent, whorls about 8, less inflated, spire high, body whorl inflated (Fig. 3). Suture shallow. Shell surface smooth, varices on whorls here and there. Yellowish-brown in colour, with red-brown fine spiral striae and irregularly red-brown longitudinal striae and with thin periostracum. The aperture wide and large, outer lip thin, simple, columella concave, without umbilicus.

#### Lacuna carinifera

(A. Adams, 1853)

861470D1-F12C-5775-84B7-45FB70553CE4

##### Native status

Lives on intertidal sandy mud, algae.

##### Distribution

China, Japan.

##### Notes

*Stenotislois* (Yen, 1936) is very similar to *Lacunacarinifera* (A. Adams, 1853), but the aperture is wider, with angulate periphery on the underside of the outer lip, the umbilicus more open. This study thinks *Stenotislois* (Yen, 1936) is the larva of *Lacunacarinifera* (A. Adams, 1853) and is a synonym.

##### Diagnosis

**Figure 4.**
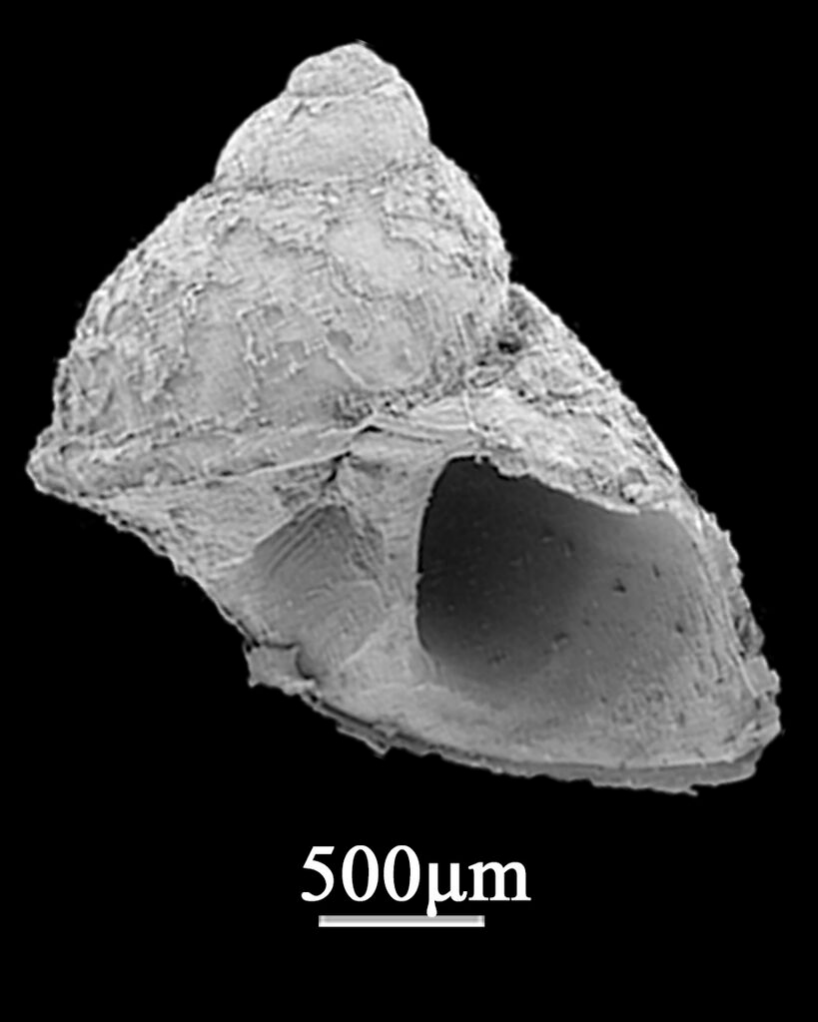
*Lacunacarinifera* (A. Adams, 1853).

Shell minute (4.3±0.76 mm in length, 1.2±0.26 mm in width), conical, depressed or auricular (Fig. 4). Whorls 4, inflated. Spire low; body whorl large, sudden expansion of width, slightly tilted. Shell thin, smooth, except for weak growth lines, with tawny periostracum. Keel round the body whorl, with grooved suture. Aperture large, pyriform, oblique, with angulate periphery on the underside of the outer lip, inner lip thick. Umbilicus open. Protoconch usually eroded.

#### Barleeia angustata

Pilsbry, 1901

2086D1E5-DB83-5F86-A597-6028BC5F9A4A

##### Native status

Lives on algae in intertidal and sublittoral zones.

##### Distribution

China, Japan, Australia.

##### Notes

The species originally belonged to Rissoidae. *Rissoinadunedini* Grabau & S. G. King, 1928 and *Rissoinanelsoni* Grabau & S. G. King, 1928 were the synonyms of this species (Zhang et al. 2016). There are shell colour variations, lightly coloured forms with distinct brown spiral bands in warm waters.

##### Diagnosis

**Figure 5.**
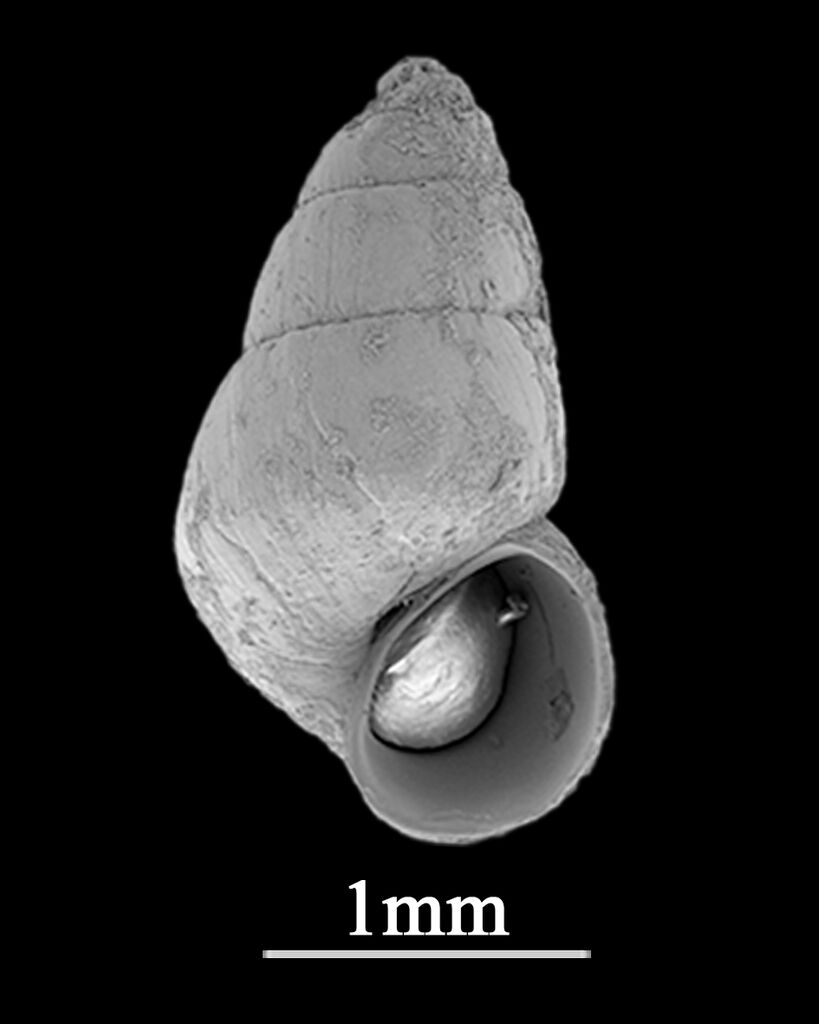
*Barleeiaangustata* (Pilsbry, 1901).

Shell minute (2.5±0.18 mm in length, 1.3±0.09 mm in width), elongate conical, solid (Fig. 5). Whorls about 5, with high spire, periphery weakly angulate in immature individuals, round when matured. Suture distinct. Shell glossy and smooth, colour uniformly reddish-brown in northern localities. Protoconch with several pits on the surface. Aperture small, peristome simple, rounded-ovate, outer lip thin, the columellar and parietal margins somewhat thickened. No umbilicus.

#### Barleeia sp.

C6F116CE-8E93-5CD0-9FEC-FF2D28FAB356

##### Native status

Lives on algae in intertidal and sublittoral zones.

##### Distribution

China

##### Notes

*Barleeia* sp. very similar to *Barleeiaangustata* (Pilsbry, 1901), but the aperture is open.

##### Diagnosis

**Figure 6.**
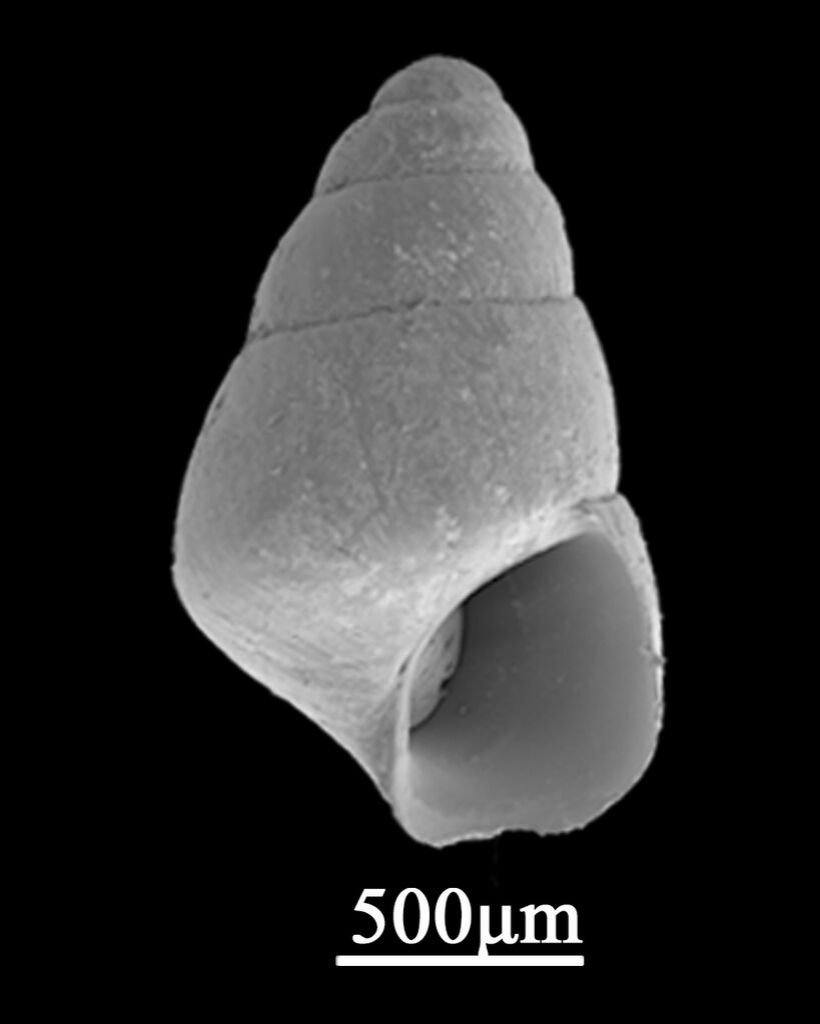
*Barleeia* sp.

Shell minute (1.2±0.42 mm in width, 1.8±0.13 mm in length), conic to ovate-conic, dark brown, smooth or with weak axial microsculptures and rather solid, periphery weak convex to angled (Fig. 6). Whorls 4, suture prominent and deep. Protoconch dome-shaped, smooth, with a diameter of about 300 μm, some sediment attached to it, with several pits on the surface. Apical angle around 45°. Aperture oval with a simple peristome, the posterior of outer lip in contact with the body whorls. Operculum, not a canal; inner lip attaching to the previous whorl, but not contacted with outer lip, not umbilicate and anterior canal; outer lip thin, anterior prosocline.

#### Iravadia elegantula

A. Adams, 1861

B7E41BEF-EC37-5BE9-BEDB-3230469A77DF

##### Native status

Brackish water, on muddy flats and under rocks in estuary at river mouth.

##### Distribution

Korea, China, Japan.

##### Notes

The *Iravadia* includes the subgenera *Fluviocingula* Kuroda & Habe, 1954, *Pseudomerelina* Ponder, 1984 and *Pseudonoba* O. Boettger, 1902. The original description from the species *Onobaelegantula* A. Adams, 1861. At present, the *Iravadiaelegantula* (A. Adams, 1861) has been accepted as *Fluviocingulaelegantula* (A. Adams, 1861).

##### Diagnosis

**Figure 7.**
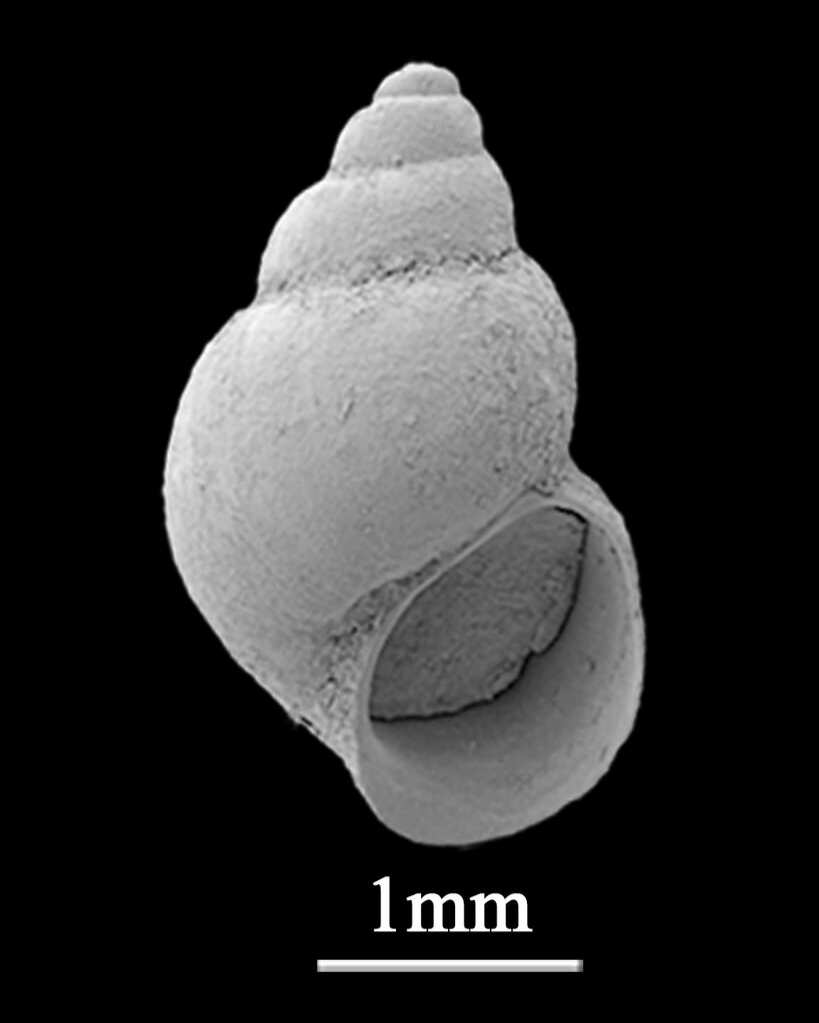
Iravadia (Fluviocingula) elegantula A. Adams, 1861.

Shell minute (3.0±0.22 mm in length, 1.6±0.14 mm in width), elongate conical, stout (Fig. 7). Whorls 5, inflated, with deeply impressed suture. Whorls increasing gradually in size. Surface of shell glossy dark brown at apical and yellowish-brown at abapical part, sculptured with many fine spiral lirae and fine growth lines. Periphery of body whorl rounded, base of whorl gradually curved. Protoconch is small, depressed dome-shape. Aperture oval and weakly angled both up and down, peristome somewhat thickened and broad down region, outer lip rounded. Umbilicus narrow and very shallow.

#### Stenothyra glabra

A. Adams, 1861

C33ADBFA-E72F-52F4-9B3D-DF60360F7946

##### Native status

Inhabiting the surface of mud flats or attaching to the under surface of floating leaves in freshwater estuaries.

##### Distribution

Yellow Sea and Bohai Sea of China, Korea and Japan.

##### Notes

The type locality of *Stenothyraglabra* A. Adams, 1861 is “estuary of the Pei-ho, North China”, which is on the coast of the Bohai Sea. One of the localities in this study, Yellow River Estuary, is adjacent to the type locality. Moreover, the shells exhibit remarkable similarity in terms of size, shape, and microsculpture when compared to the descriptions provided by Adams (1861), Yen (1939), Yen (1942), and Kantor and Sysoev (2006), although the available photograph of the holotype is lacking (Agatsuma et al. 2005). We believe that specimens collected in this study belong to common species from the coasts of the Yellow Sea and Bohai Sea in China and are conspecific with the type material.

##### Diagnosis

**Figure 8.**
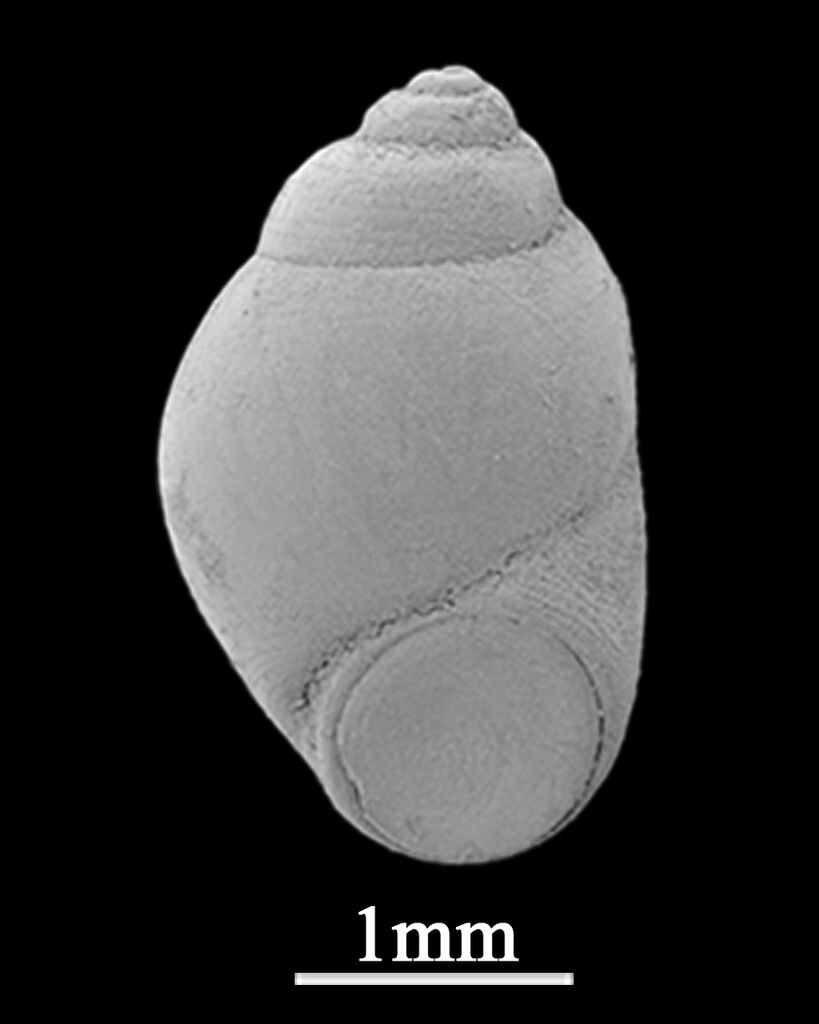
*Stenothyraglabra* A. Adams, 1861.

Shell minute (2.1±0.14 mm in length, 1.5±0.07 mm in width), ovate-conic, rather thick, dorso-ventrically compressed, with rounded to angled inflation of last whorl (Fig. 8). Up to 5 whorls including protoconch, with less convex whorls and sutures moderately deep. Surface smooth, yellowish-brown, sculpture not dotted lines, but continuous spiral grooves. The aperture abruptly descending, contracted and near circular; peristome continuous, showing a weak triangular area above; outer lip round with marked grooves. Operculum ovate, yellowish, translucent, with very weak angulation aligning with posterior apex of aperture; exterior surface with central paucispiral nucleus close to the inner lip. Protoconch dome-shaped, smooth, 1-3/4-2 whorls; some pits in the first whorl.

#### Assiminea estuarina

Habe, 1946

8B588383-D075-5925-9124-91DCB8A8FE8D

##### Native status

Lives on mud bottoms in brackish water areas of estuaries.

##### Distribution

China, Japan.

##### Notes

The genus *Assiminea* H. & A. Adams, 1865 is cosmopolitan, but most of them are from the Indo-pacific Region. *A.estuarina* is rarely found in China. The shell of this species is similar to *A.hiradoensis* Habe, 1942, but there are some differences between them. Each whorl of *A.estuarina* is more convex than *A.hiradoensis*. The height of the spire of *A.hiradoensis* is higher.

##### Diagnosis

**Figure 9.**
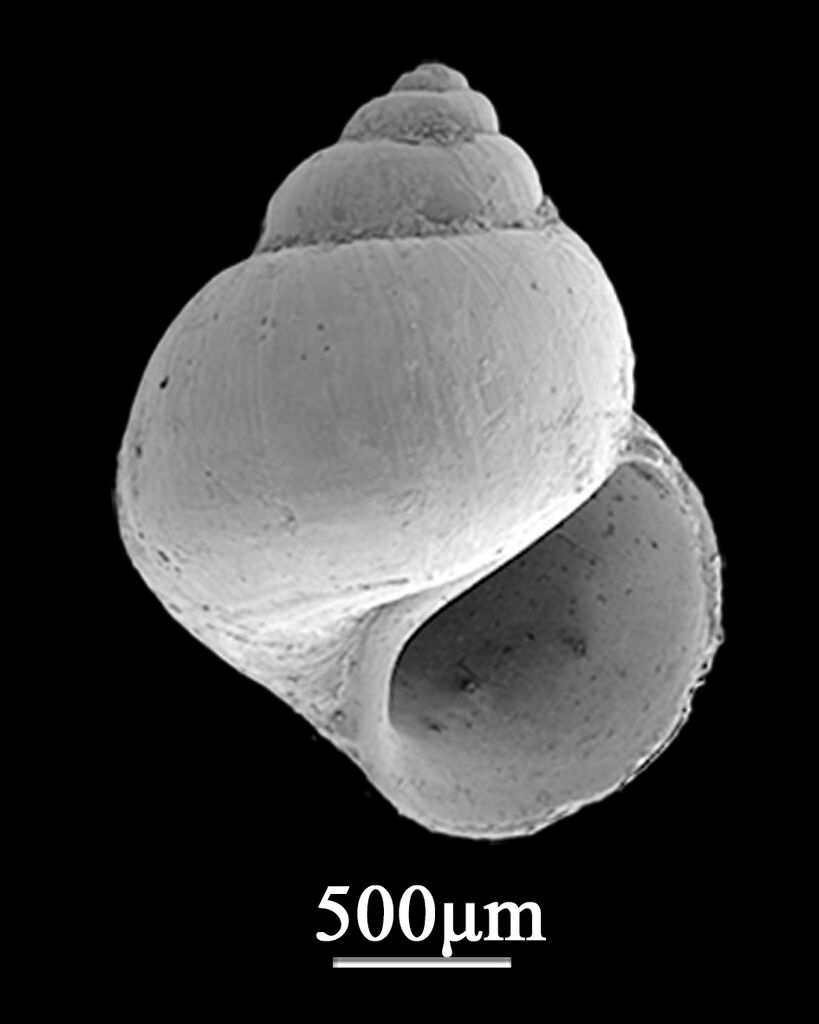
*Assimineaestuarina* Habe, 1946.

Shell minute (2.2±0.36 mm in length, 1.5±0.08 mm in width), globose-conic, solid (Fig. 9). Whorls 5, spire low, each whorl weakly convex in spire, body whorl large and more convex, with impressed suture. Surface smooth, with many fine axial growth threads, yellowish, with 2 brown bands on body whorl. Aperture large, simple, ovate, columellar lip of aperture pale brown, but inner lip dark purplish-brown. Early whorls of teleoconch without spiral ribs.

#### Alvania concinna

A. Adams, 1861

469B64F9-9A91-5F3F-99AC-FFA2D9DF2147

##### Native status

Lives in intertidal areas in sheltered bays on seaweed.

##### Distribution

China, Japan and Korea.

##### Notes

The trait of the genus *Alvania* Risso, 1826 is that the teleoconch is ovate-conic to elongate-conic, with clathrate sculpture or spiral sculpture (Adams 1850). The changes of morphological characteristics and surface colour are relatively large, the synonym *Hydrobiaplicosa* E. A. Smith, 1875 is a northern form of this species (Okutani 2017).

##### Diagnosis

**Figure 10.**
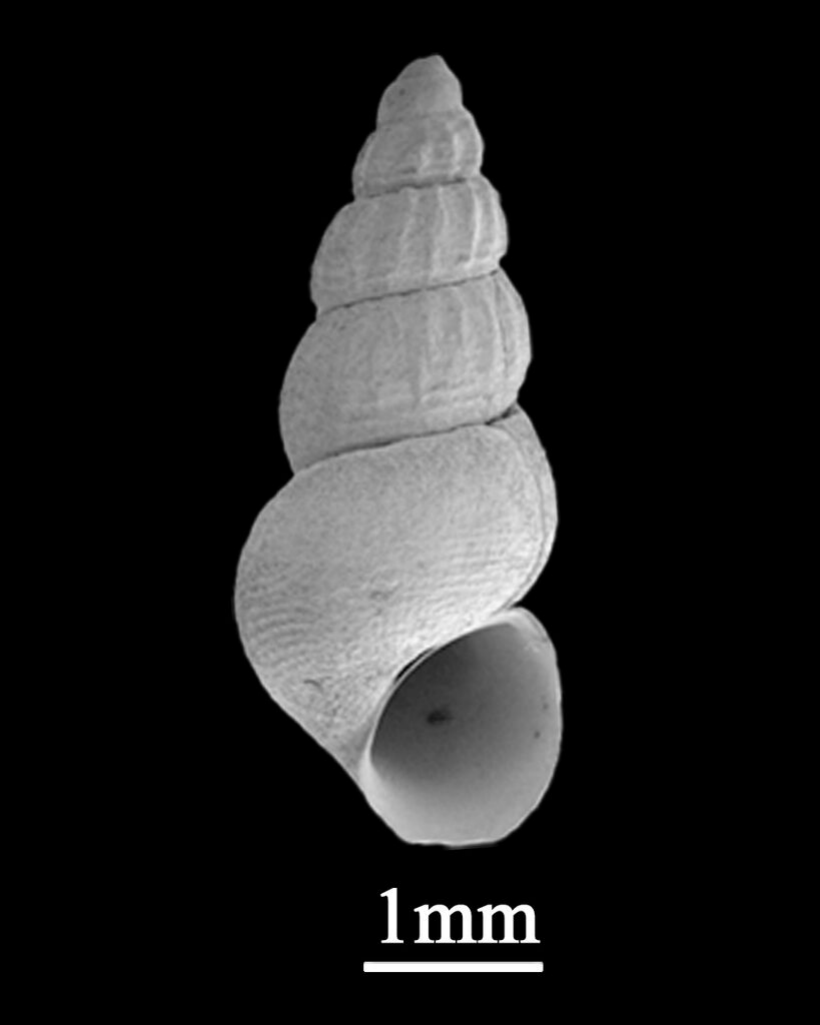
*Alvaniaconcinna* A. Adams, 1861.

Shell minute (4.5±0.41 mm in length, 2.4±0.11 mm in width), elongate conical (Fig. 10). Whorls about 6, slightly convex, with elevated spire, deeply sutured. Shell surface reddish-brown, sculptured with many spiral ribs and strong axial ribs; axials tend to weaken on body whorls in populations in temperate waters. Aperture simple, ovate, outer lip thin, without varix, the upper part of outer lip slightly angular. Apex generally not strongly tilted in species with paucispiral protoconch. No umbilicus.

#### Pseudoliotia pulchella

Dunker, 1860

6926DD7D-168C-5CDC-95D1-512CACAA70B2

##### Native status

Under rocks on sandy gravel bottoms in intertidal zones in sheltered areas.

##### Distribution

China, Japan.

##### Notes

*Pseudoliotia* Tate, 1898 is characterised by a depressed conical shell with strong axial and spiral ribs on its surface. *P.pulchella* is similar to *P.astericus*, but much larger, lower in shell height and with relatively weak axial ribs.

##### Diagnosis

**Figure 11.**
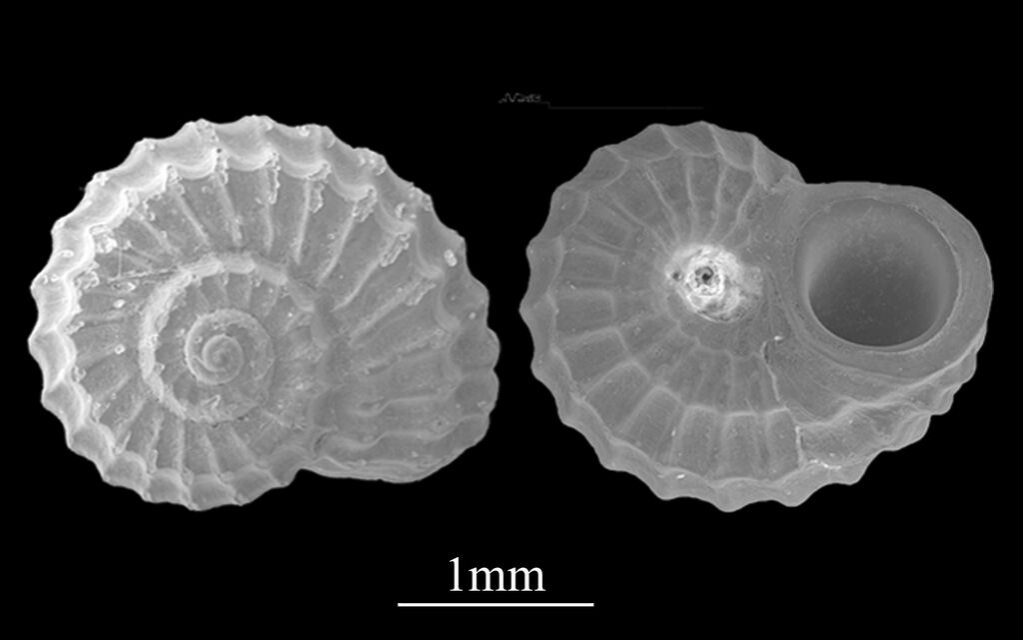
*Pseudoliotiapulchella* (Dunker, 1860).

Shell minute (1.3±0.19 mm in length, 3.1±0.16 mm in width), depressed conical, round pie, solid (Fig. 11). Whorls about 5, spire very low, body whorl large, the spiral layers are almost in the same plane, whorl periphery appears markedly truncate with a deep concave sulcus between the two peripheral keels. Suture distinct, grooved. Surface sculptured with thick spiral cords and many strong axial ribs, white or covered with yellow crust, thin sculpture lines on the sides of the whorl. Protoconch smooth. Aperture thickened, ovate, declining. Umbilicus deep.

#### Peasiella habei

D. Reid & Mak,1998

270BBA57-52C9-5B8F-B229-032F63C3C26F

##### Native status

The species is abundant in crevices and amongst barnacles in the middle and upper eulittoral zone, on sheltered and moderately exposed rocky shores; on exposed shores, it shows a preference for surfaces protected from wave action.

##### Distribution

China, Korea, Taiwan, Japan.

##### Notes

This species is variable in conspicuous features of the shell including colour, spire profile and sculpture, but consistent characteristics are the row of dark spots above the periphery, which extend on to the pale peripheral keel, the darker and often black spire whorls and the prominent keel at the periphery.

##### Diagnosis

**Figure 12.**
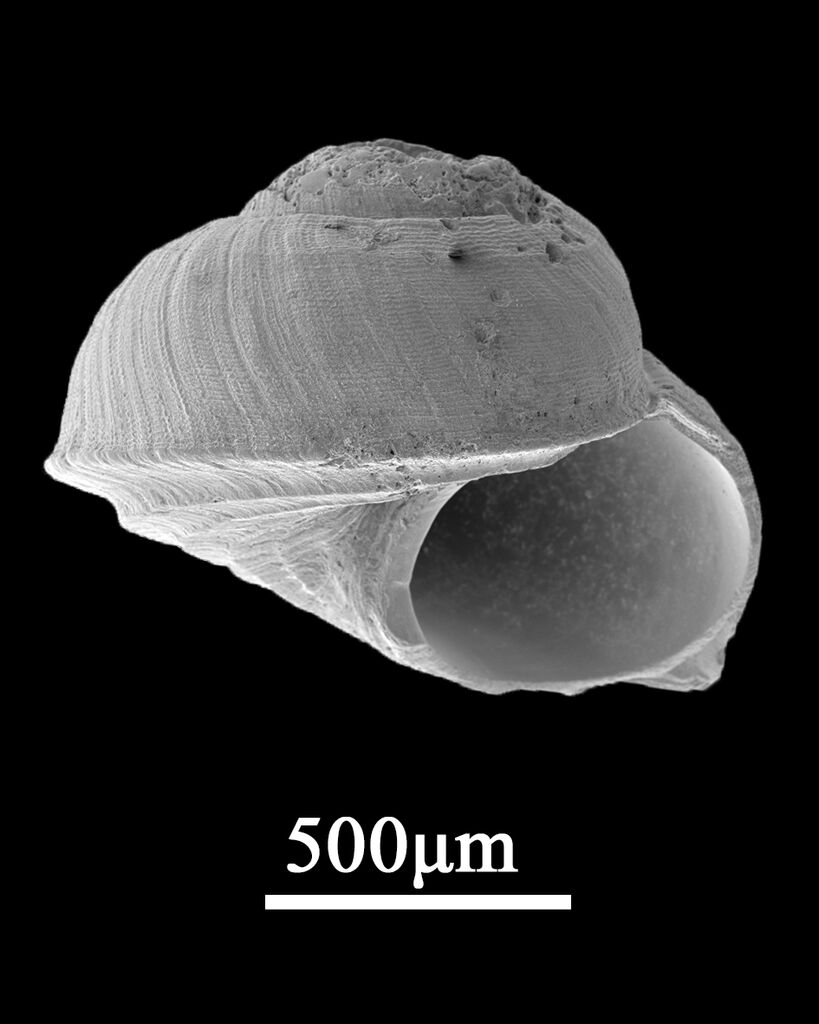
*Peasiellahabei* D. Reid & Mak,1998.

Shell minute (3.0±0.28 mm in length, 2.4±0.09 mm in width), depressed conical, thick, stout; outline domed (Fig. 12). Whorls 3, whorls almost flat-sided or rounded or slightly shouldered; body whorl large with sharply angulate periphery; teleoconch whorls usually smooth, with spiral microstriae. Base flat to slightly rounded, with about 4 spiral ribs. Dark brown with irregular white spots. Suture impressed; peripheral keel prominent, often a projecting flange, rarely slightly undulating. Umbilicus usually narrow; columella narrow, curved at base. Protoconch usually eroded. Aperture ovate, outer lip thin and bottom angular, inner lip slightly thick and the upper part not connected with outer lip.

#### Acteocina fusiformis

A. Adams, 1850

47114F17-AC81-5C95-957F-ED39298C8BA0

##### Native status

Found on sandy and mud bottoms in intertidal seagrass bed.

##### Distribution

Bohai Sea in China, Japan, South African.

##### Notes

Shell cylindrical or fusiform, spire conspicuous, apex papillated, suture channelled, columella callous, with a single fold in the genus *Acteocina* Gray, 1847 (Adams 1850). The division of the genus *Acteocina* is problematic in systematics. Okutani (2017) divided it into the family Cylichnidae, but it was divided into Tornatinidae in WoRMS.

##### Diagnosis

**Figure 13.**
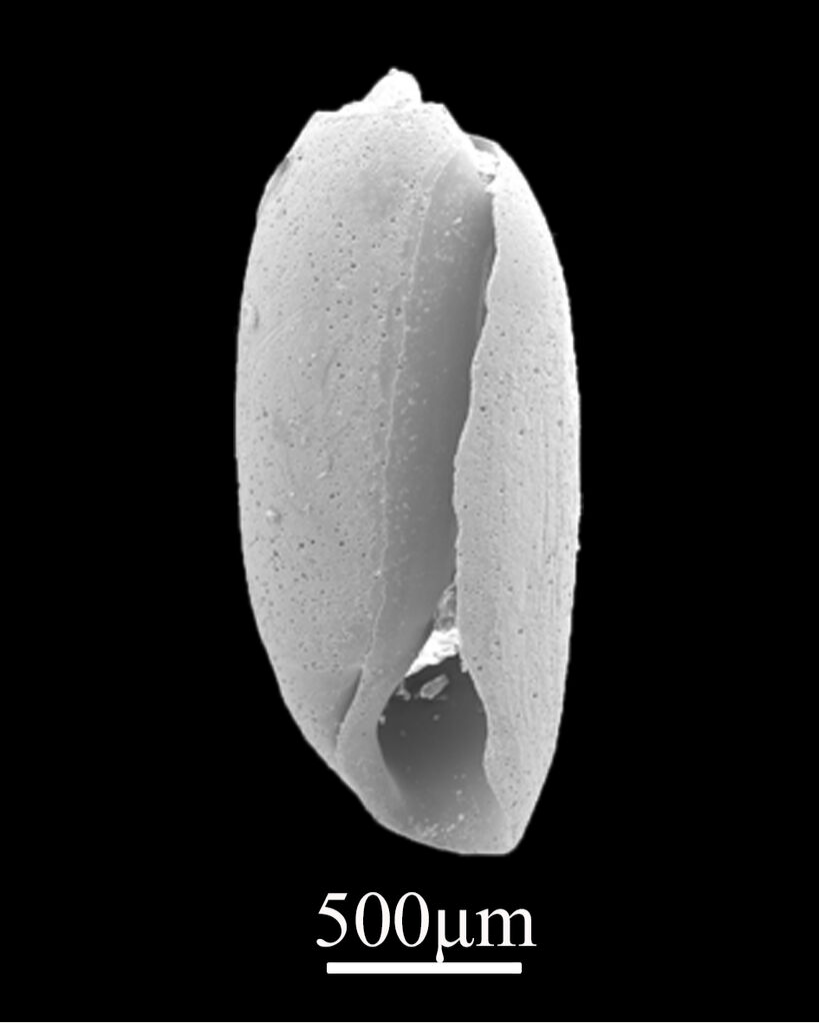
*Acteocinafusiformis* A. Adams, 1850.

Shell minute (2.5±0.15 mm in length, 0.9±0.08 mm in width), cylindrical to fusiform, thick, solid, with fine and indistinct spiral grooves (Fig. 13). Surface white, smooth, slightly corroded. Whorls about 3, spire small, body whorl was slightly constricted on the upper part. Shoulder acutely angulated. Protoconch prominently projecting. Suture oblique. Columella stout, with a fold above.

#### Pyrunculus tokyoensis

Habe, 1950

D3B4522E-9CB4-5346-A4BE-F860BCC85B98

##### Native status

Found on sandy and mud bottoms in intertidal seagrass beds.

##### Distribution

China and Japan.

##### Notes

*P.tokyoensis* is similar to *P.phiala*, but differs from *P.phiala* in having distinct spiral grooves overall.

##### Diagnosis

**Figure 14.**
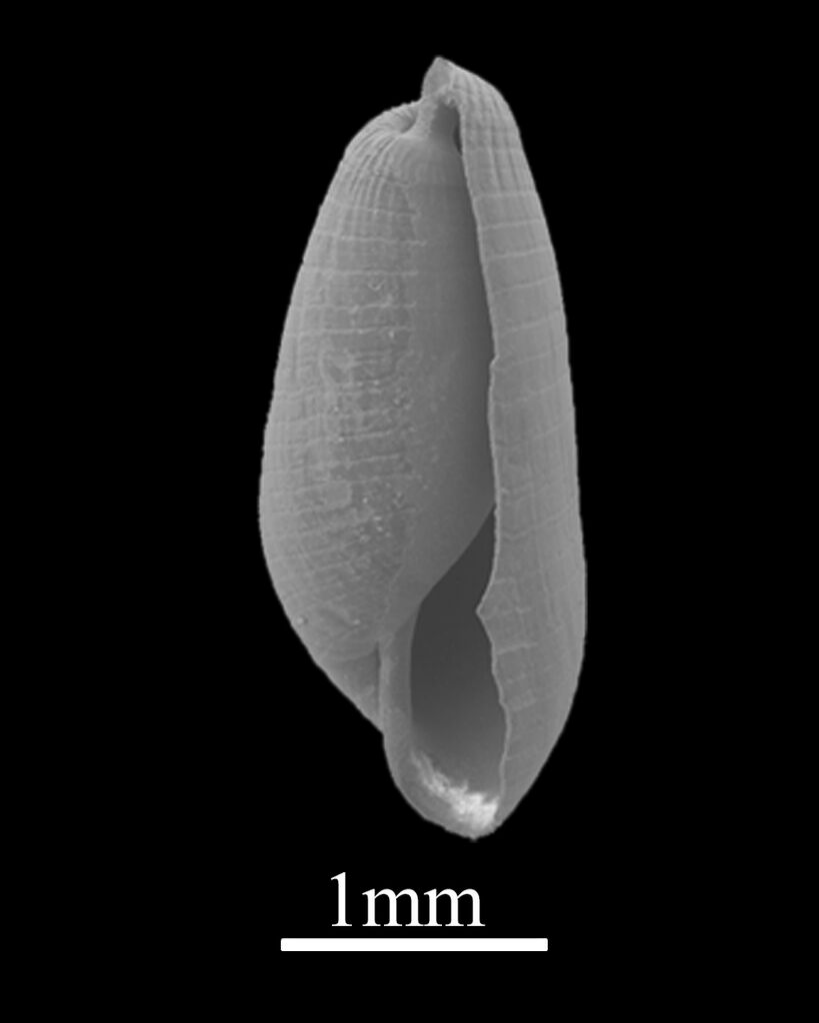
*Pyrunculustokyoensis* Habe, 1950.

Shell minute (3.0±0.34 mm in length, 1.3±0.11 mm in width), pyriform, thin, white, polished (Fig. 14). Apex narrowly perforated with rounded margin. The upper end of the body spiral shrinking and the lower end expanding, covered with spiral grooves overall, axial grooves distinct on upper extremity. Columella with fold. Outer lip thin.

#### Brachystomia bipyramidata

Nomura, 1936

164791E4-10F8-54E5-A440-7A3D6ABA09CB

##### Native status

Sucking body fluid of *Crassostreagigas* on rocks in intertidal zones.

##### Distribution

North of China, Japan.

##### Notes

The genus *Brachystomia* Monterosato, 1884 is very similar to *Odostomia* spp. in shell characters, but differs in the protoconch turning down on the teleoconch apex, attaching to other molluscs when alive (Okutani 2017).

##### Diagnosis

**Figure 15.**
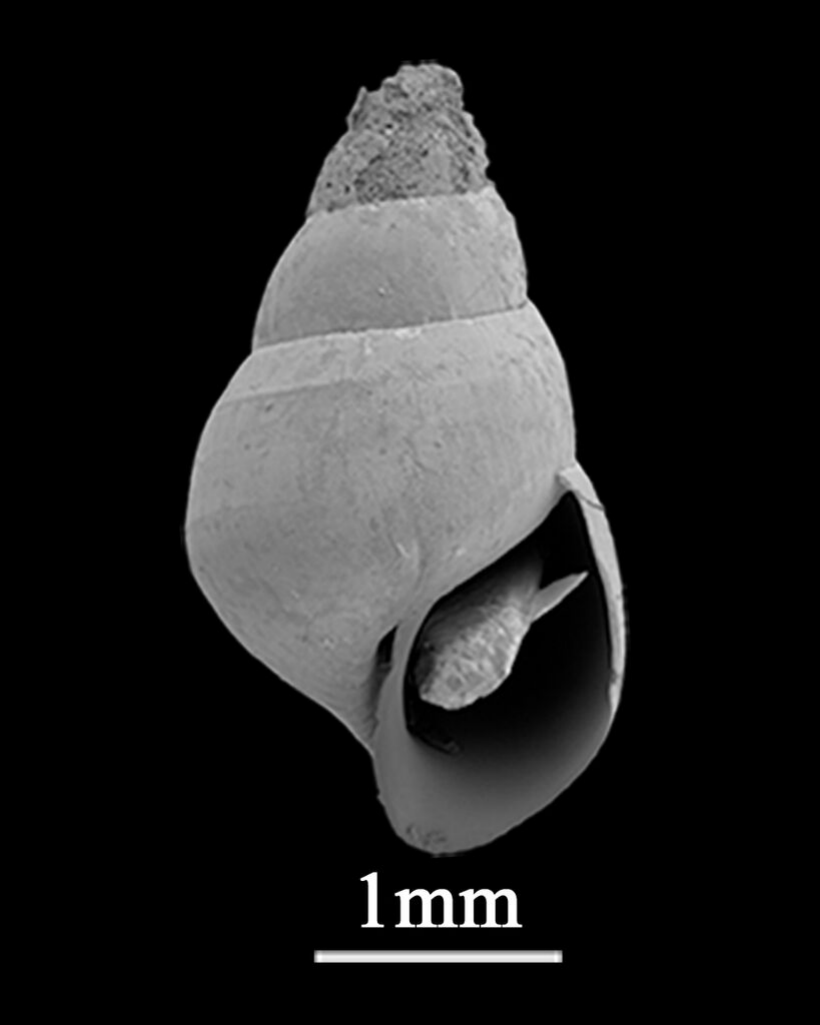
*Brachystomiabipyramidata* (Nomura, 1936).

Shell minute (3.2±0.27 mm in height, 1.5±0.34 mm in width), oval conical, moderately thick, translucently milky-white, solid (Fig. 15). Whorls about 5, each spiral slightly convex, the height of the body whorl is larger than spire. Surface covered with yellowish-brown periostracum. Suture distinct, grooved. Growth lines coarse and flexuous. Aperture ovate. Columella with a weak fold. Outer lip margin with a shallow posterior sinus.

#### Odostomia subangulata

A. Adams, 1860

A45FC798-E5B6-5B07-8B7A-B0891EABDE8A

##### Native status

Sucking body fluid of *Crassostreagigas* on rocks in intertidal zones.

##### Distribution

North of China, Japan.

##### Notes

*Megastomiatenera* (A. Adams, 1860) very similar to *Odostomiasubangulata* A. Adams, 1860 in shell characters, but differs in having palatal ridges in the inner lip.

##### Diagnosis

**Figure 16.**
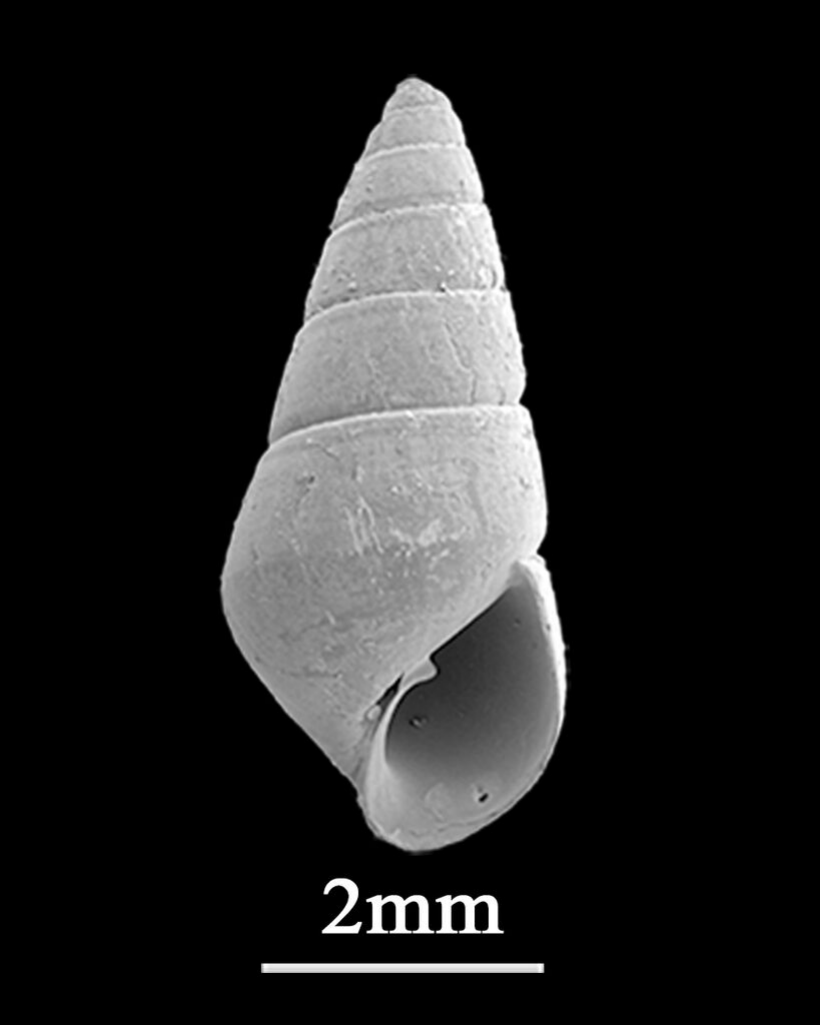
*Odostomiasubangulata* A. Adams, 1860.

Shell minute (5.0±0.12 mm in height, 2.3±0.03 mm in width), conical, milky-white, moderately thick, solid (Fig. 16). Whorls about 6, each whorl nearly flat-sided, shoulder of each whorl feebly angulate. Sutures distinct, grooved. Protoconch immersed in first teleoconch whorl, rotated right. Umbilicus narrowly opened. Aperture simple, long-ovate, columella with fold, outer lip thin, curved.

#### Turbonilla osyuensus

Nomura, 1936

503E0FD2-F9A9-5F3F-A694-8B44FC4363D2

##### Native status

Found on sandy mud bottoms of seagrass beds.

##### Distribution

Bohai Sea of China, Japan.

##### Notes

*Chemnitziaosyuensis* (Nomura, 1936) is the type specimen and a synonym. Very similar to *T.gracilenta*, but differs in the protoconch rotated right (Higo and Goto 1993).

##### Diagnosis

**Figure 17.**
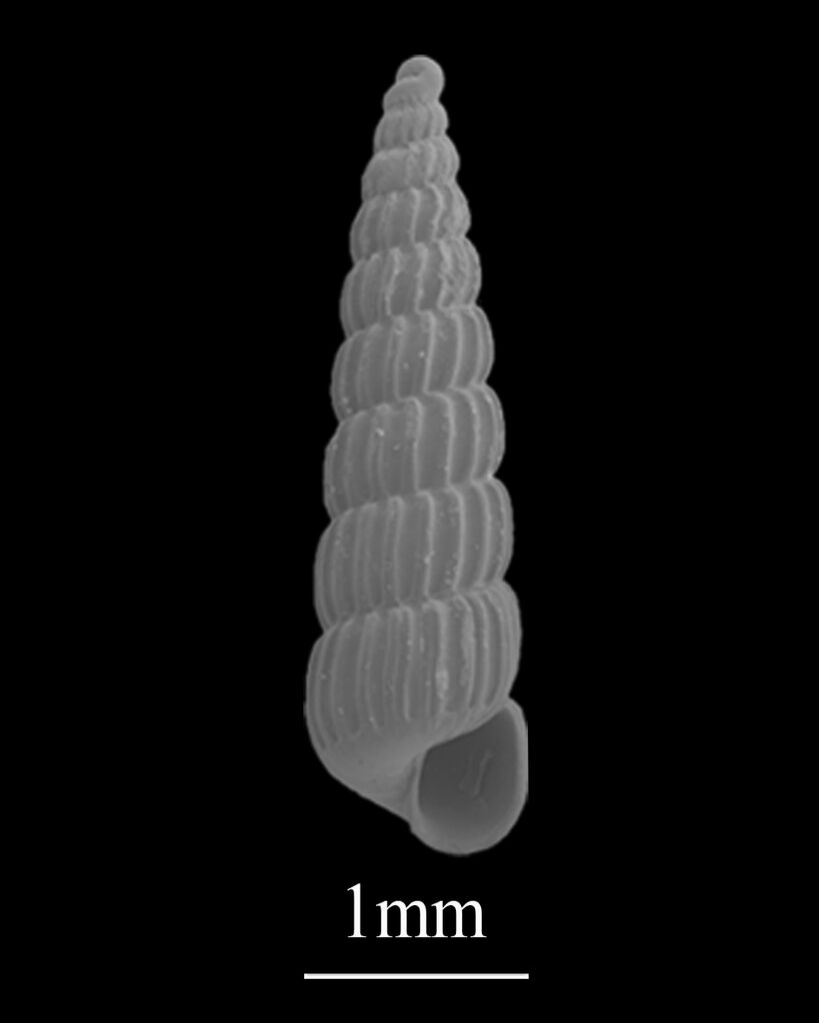
*Turbonillaosyuensus* Nomura, 1936.

Shell minute (3.8±0.13 mm in height, 1.0±0.21 mm in width), towered, moderately solid, white (Fig. 17). Whorls about 10, each whorl with convex sides. Sutures constricted. Axial ribs oblique and straight, terminating at periphery. Interspaces of axial ribs twice as wide as ribs, the axial rib of the upper whorl is aligned with the interspace of the lower whorl. Aperture simple, upper of the outer lip with angular, columella with no folds, basal lip circle. The apex of the protoconch is composed of two parts of different sizes.

#### Rissoella elatior

Golikov, Gulbin & Sirenko, 1987

480926B2-A16A-5812-AF58-874875D68BED

##### Native status

Living on seagrass.

##### Distribution

China, Japan, Russia.

##### Notes

The shell characteristics of the genus *Rissoella* are thin; vitreous and soft parts can be seen through it. As some species are similar in morphology, many species are misidentified or undescribed. In this study, our sampled material agrees with the original description of this species (Golikov et al. 1987), as well as the study from Siaden et al. (2019).

##### Diagnosis

**Figure 18.**
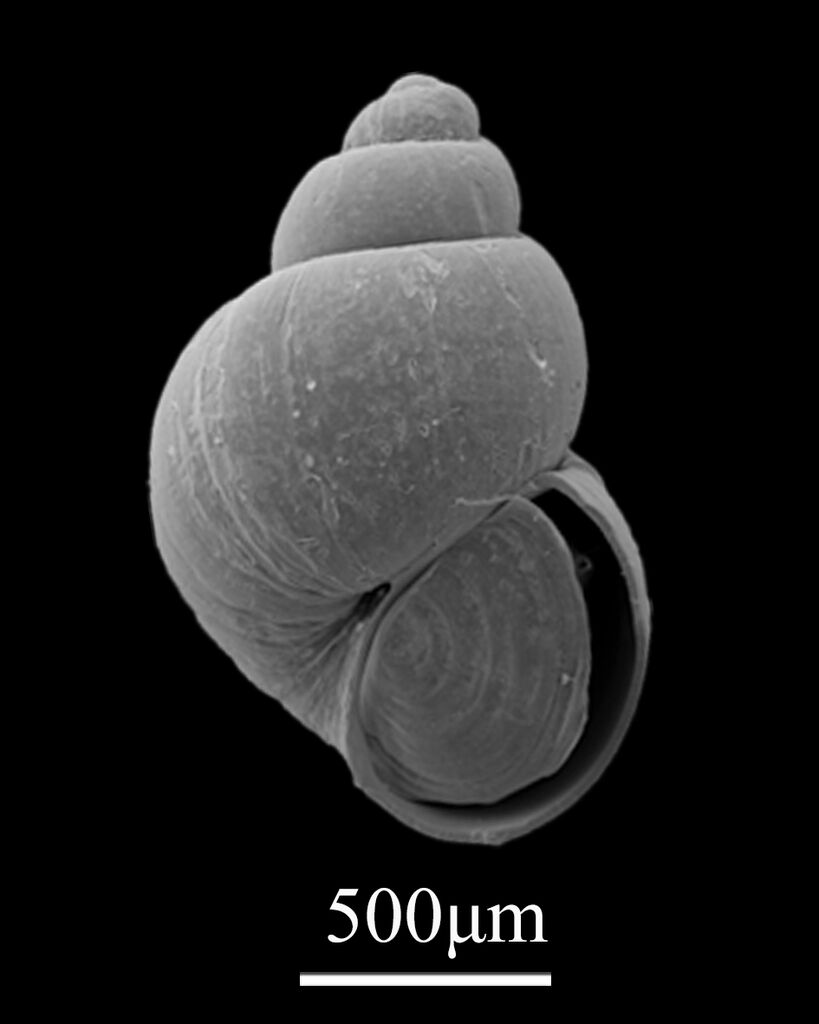
*Rissoellaelatior* (Golikov, Gulbin & Sirenko, 1987).

Shell minute (1.5±0.12 mm in height, 0.88±0.06 mm in width), elongate oval, thin, vitreous, fragile, translucent (Fig. 18). Whorls about 4, each whorl rather convex, spire about 25% of shell height. With deep sutures. Surface almost smooth, the axial growth lines of body whorl shrinking at base. Protoconch smooth, low. Aperture simple, ovate. With narrow umbilicus.

#### Lasaea undulata

A. A. Gould, 1861

EDBE1EF2-F916-5E7D-BCAB-0D1011D49037

##### Native status

Attached to the byssus of the septifer and the roots of seaweeds between tidal marks.

##### Distribution

Yellow Bay (Shandong Province) of China, Japan.

##### Notes

*Lasaea* consists of small pelecypod species, the largest of which attains a size of about 8 mm in length; adult specimens of the smaller species are about 2 mm long. In general, these shells are quadrangular in outline; they are equivalved, but inequilateral, the anterior end being the longer (Keen 1938). Most, if not all, species of *Lasaea* are nestlers, finding shelter in dead barnacle tests and amongst the holdfasts of seaweeds. It is cosmopolitan, but only *L.undulata* is found in China.

##### Diagnosis

**Figure 19.**
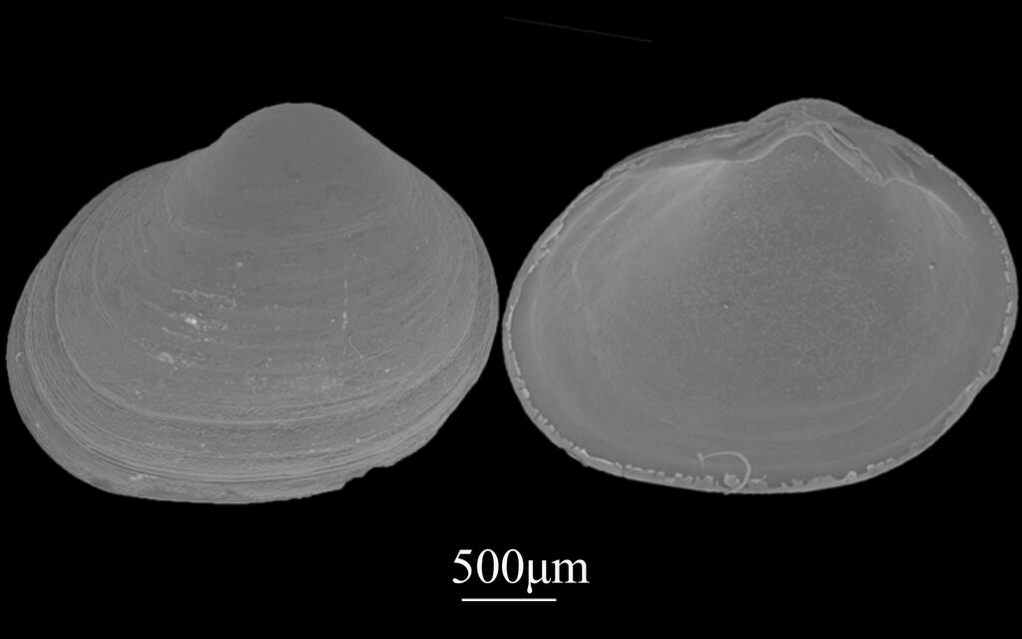
*Lasaeaundulata* (A. A. Gould, 1861).

Shell small (2.7±0.15 mm in length, 1.9±0.11 mm in height), elongate-ovate, thin, fragile (Fig. 19). Surface purplish-red, ornamented with concentric growth striae. Umbo prominent, situated slightly behind the mid-point of the dorsal margin. Break located near posterior margin. Hinge plate very strong, with one cardinal socket, with one anterior lateral cardinal tooth and two posterior lateral cardinal teeth of different sizes in right valve.

#### Alveinus ojianus

Yokoyama, 1927

1C923C44-1F9A-5CD2-ADCD-723B98EE4A34

##### Native status

Found on sandy mud bottoms in upper sublittoral zones.

##### Distribution

Yellow Sea and Bohai Sea of China, southern Japan.

##### Notes

*Spaniodon* Reuss, 1867 was considered a senior synonym of *Alveinus* Conrad, 1865, containing only *Alveinusmiliaceus* (Issel, 1869) and *Alveinusojianus* (Yokoyama, 1927) in WoRMS. *A.miliaceus* is very similar to *A.ojianus* in shell characters, but differs in the cardinal tooth. The cardinal teeth bifurcate into two lamellae (Conrad 1865).

##### Diagnosis

**Figure 20.**
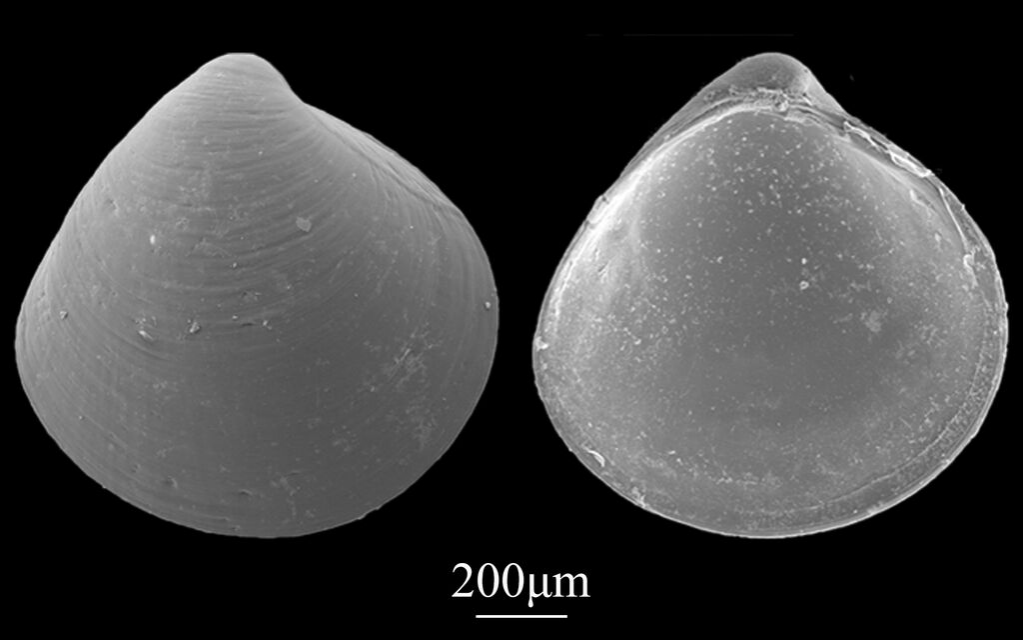
*Alveinusojianus* (Yokoyama, 1927).

Shell minute (1.1±0.17 mm in length, 1.0±0.22 mm in height), triangular-ovate, inflated, equivalved (Fig. 20). Umbo prominent, almost situated at the middle of the dorsal margin. Anterior and posterior ends round. Surface smooth and yellowish, ornamented by densely-spaced co-marginal lamellae. Hinge plate with one anterior and one posterior cardinal tooth in the left valve and one cardinal tooth in the right ligament external parivincular, with small resilium.

#### Bentharca sp.

39CD8A30-AB3D-5E09-A01E-3188B448E6F3

##### Native status

Found on sandy mud bottoms in upper sublittoral zones.

##### Distribution

Bohai Sea of China.

##### Notes

This species is very similar to *Bentharca* spp. in shell characters including radial riblets and is covered with lamellate epidermis at interstices, cardinal plate and umbo.

##### Diagnosis

**Figure 21.**
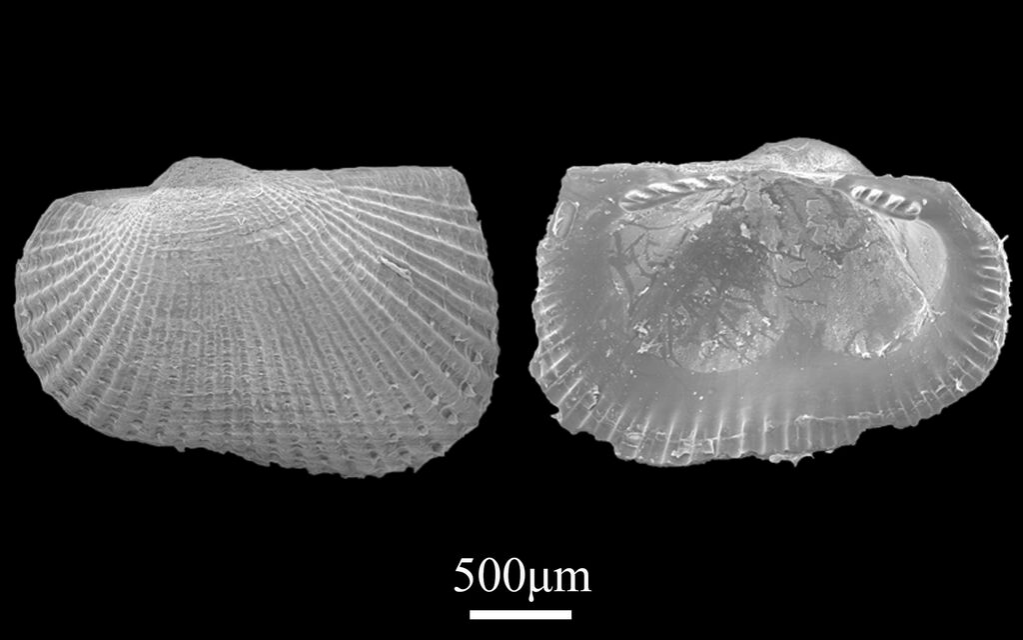
*Bentharca* sp.

Shell minute (2.3±0.02 mm in length, 1.5±0.04 mm in height), elongate squarish, thin, moderately inflated (Fig. 21). Antero-posterior margin round, anterior narrower and shorter than posterior; posterior hardly oblique, sides weakly angulated at upper part. Umbo corroded, situated in front of dorsal margin. Surface with 33 radial riblets of various prominence and strong concentric ribs near the umbo; covered with lamellate epidermis at interstices; inner surface margin is toothed. With a depression at the ventral margin. Cardinal plate with 3 teeth on anterior arc and 4 on posterior arc.

## Analysis

A total of 20 species were described in this survey (Table 2). Out of the 20 specimens, identification was verified by barcoding for 13 specimens whose sequences have been deposited in GenBank. These can be assigned to 16 families, four orders (Vetigastropoda (1), Caenogastropoda (10), Heterobranchia (6) and Autobranchia (3)) and two classes (Gastropoda (17), Bivalvia (3)).

## Discussion

**Figure 22.**
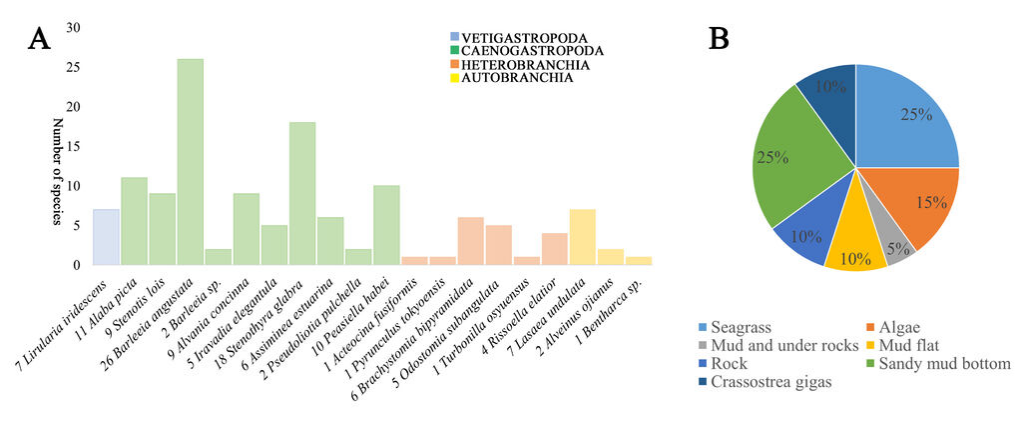
The statistical chart of micromolluscs diversity and habitat. **A** The numbers in front of the species names indicate the number of collected specimens. Different colours represent different orders. **B** The percentage of species in different habitats. Different colours represent different habitats.

This study described the diversity of common micromolluscs from the Yellow Sea and Bohai Sea in China. A total of 20 species were collected, belonging to 16 families and 16 genera of Gastropoda and 3 families and 3 genera of Bivalve, respectively. The statistics show that Caenogastropoda has the highest species diversity, which should be attributed to two essentially micromollusc groups, Rissooidea and Truncatelloidea (Fig. 22A). However, Vetigastropoda have the least diversity. This was due to the micromolluscs of Vetigastropoda being difficult to collect, as many in the families, Solariellidae, Liotinidae, Crosseolidae, Skeneidae, Anatomidae and Scissurellidae inhabited the subtidal zone (10-500 m depth).

In terms of morphology, this study analysed the surface characteristics of the shells in detail by taking SEM images of the shells and carried out morphological identification and description, but there are still many undetermined species and, due to the limited amount of sample, a further morphological study could not be carried out. Meanwhile, this also means that there are still many micromolluscs to describe.

According to the survey, it is found that most of the micromolluscs inhabit seagrass (25%), sandy mud bottom (25%), algae (15%), mud and under rocks (5%) and others habitats (respectively 10%) (Fig. 22B). A total of about 40% of them living on seagrass and algae may be associated with their feeding and further refuging habits to reduce predation pressure (Holthuis 1995). Interestingly, *B.bipyramidata* and *O.subangulata* from Pyramidellidae were associated with *Crassostreagigas*, which are often considered parasites or symbionts of bivalves (Fretter and Graham 1986), while some Pyramidellidae species, such as *Odostomiaturrita*, *Odostomiaacuta* and *Spiralinellaspiralis*, were confirmed to associate with tubeworms from polychaetes (Høisæter 2014). Some 25% of collected samples were from sandy mud bottoms, because most of them contained only a shell. There are also fewer micromolluscs inhabiting mud flat and rocks, which may indicate that shellfish like to choose the best habitat in terms of habitat space and this selectivity is related to the geography of the sea area. Factors such as location, bottom deposition type, wave disturbance, abundance of food and seasonal changes are closely related.

## Supplementary Material

XML Treatment for Lirularia iridescens

XML Treatment for Alaba picta

XML Treatment for Lacuna carinifera

XML Treatment for Barleeia angustata

XML Treatment for Barleeia sp.

XML Treatment for Iravadia elegantula

XML Treatment for Stenothyra glabra

XML Treatment for Assiminea estuarina

XML Treatment for Alvania concinna

XML Treatment for Pseudoliotia pulchella

XML Treatment for Peasiella habei

XML Treatment for Acteocina fusiformis

XML Treatment for Pyrunculus tokyoensis

XML Treatment for Brachystomia bipyramidata

XML Treatment for Odostomia subangulata

XML Treatment for Turbonilla osyuensus

XML Treatment for Rissoella elatior

XML Treatment for Lasaea undulata

XML Treatment for Alveinus ojianus

XML Treatment for Bentharca sp.

## Figures and Tables

**Table 1.** Details on collection sites (Fig. 1).

Name	Abbreviation	Area and Geographic Data	Date of Collection
Tangshan	TS	38°54′47″N, 118°29′35″E	20.10.2017
Dongying	DY	37°48′49″N, 119°18′57″E	24.06.2018
Yantai	YT	37°33′34″N, 121°30′58″E	12.08.2018
Weihai	WH	37°21′35″N, 122°34′38″E	03.06.2019
Qingdao	QD	36°03′18″N, 120°19′47″E	09.07.2019
Ganyu	GY	34°51′00″N, 119°13′17″E	20.09.2019

**Table 2.** Species used in this study, species name and taxonomic ranks, GenBank accession numbers, locality of collection and Date of Collection.

		Family	Species name	Genbank	Locality	Date of Collection
Gastropoda	Vetigastropoda	Trochidae	*Lirulariairidescens* (Schrenck, 1863)	MT254075	Weihai	2018
	Caenogastropoda	Litiopidae	*Alabapicta* (A. Adams, 1861)	MT254074	Qingdao	2018
		Barleeiidae	*Barleeiaangustata* (Pilsbry, 1901)	MT254076	Weihai	2018
			*Barleeia* sp.	MT254077	Qingdao	2017
		Iravadiidae	*Iravadiaelegantula* (A. Adams, 1861)	MT240257	Dongying	2017
		Stenothyridae	*Stenothyraglabra* A. Adams, 1861	MN548735	Ganyu	2019
		Assimineidae	*Assimineaestuarina* Habe, 1946	MT240258	Weihai	2019
		Rissoidae	*Alvaniaconcinna* A. Adams, 1861	MT240259	Weihai	2018
		Tornidae	*Pseudoliotiapulchella* (Dunker, 1860)	-	Tangshan	2018
		Littorinidae	*Peasiellahabei* D. Reid & Mak,1998	MT823260	Qingdao	2019
			*Lacunacarinifera* (A. Adams, 1853)	MT823257	Weihai	2018
	Heterobranchia	Tornatinidae	*Acteocinafusiformis* (A. Adams, 1850)	-	Tangshan	2019
		Retusidae	*Pyrunculustokyoensis* Habe, 1950	-	Tangshan	2019
		Pyramidellidae	*Brachystomiabipyramidata*（Nomura,1936）	MT823256	Qingdao	2018
			*Odostomiasubangulata* A. Adams, 1860	MT254072	Qingdao	2018
			*Turbonillaosyuensus* Nomura, 1936	-	Tangshan	2018
		Rissoellidae	*Rissoellaelatior* (Golikov, Gulbin & Sirenko, 1987)	-	Tangshan	2019
Bivalvia	Autobranchia	Lasaeidae	*Lasaeaundulata* (A. A. Gould, 1861)	MT254080	Weihai	2018
		Kelliellidae	*Alveinusojianus* (Yokoyama, 1927)	-	Tangshan	2019
		Arcidae	*Bentharca* sp.	-	Tangshan	2019
